# Correspondence

**Published:** 1895-12

**Authors:** 


### CORRESPONDENCE.

Editor Journal of Comparative Medicine, etc.

Dear Sir: In your November issue I read the article on "Stable Hygiene" and the criticism of Dr. W. R. Claussen in regard to the article by Dr. Sallade. I will say that the merits of a plan of ventilation depend not only on the amount of air introduced into a stable, but on the distribution of that air. I have made some careful experiments in regard to the distribution of air in stables; have taken samples of air from the floor to roof in different parts of the room (stable), submitted these samples to chemical analysis, and find that the carbonic acid is present in about equal proportions in all parts of the room. Hence I have come to the conclusion that the law of diffusion of gases effectually prevents this separation of gases that Dr. W. R. Claussen brings as the argument. I think we ought not to fall into the error of supposing that because $CO_2$ is heavier than air, therefore the $CO_2$ derived from respiration sinks to the floor.

There seems to be a notion that the outlets should be larger than the inlets where air is to be admitted and discharged "on account of expansion of heated air," but this is not so. I like top-ventilation, and employ it whenever I can, but we must have a corresponding inlet for an outlet without, however, creating draughts upon the animals.

Respectfully,

HERBERT NEHER, D.V.S.

NEW YORK, November 14, 1895.

---

EDITOR JOURNAL OF COMPARATIVE MEDICINE, ETC.

DEAR SIR: In the November issue of the JOURNAL there seems to be quite a difference of opinion in regard to the method of stable ventilation as recommended by Dr. Sallade in the August number; that is, top ventilation.

As I am greatly interested in this subject and a firm believer in the laws of hygiene, deeming it one of the most essential points to be considered by any person who is to take the responsibilities of combating disease either in the human family or the animal kingdom, and as there can be no doubt that the best possible sanitary conditions that can be provided for a sick animal are one of the first steps toward promoting successful results of therapeutic medicine when properly applied. There is no gainsaying the fact that a very large number of practitioners of medicine place too much dependence on drugs and too little attention to the laws of nature, which has been fully proven and demonstrated by scientific research and investigation, as we see by comparing the present with the past, proving beyond a doubt that the laws of hygiene are the laws of health. One of the first essentials under this law is the very best and purest air we can provide—the principal life-giving element to all living beings.

I am of the opinion that Dr. Sallade is perfectly correct on the question of top ventilation to allow the escape of the foul and polluted air of the stable, but should have suggested in addition to his plan a way whereby the ingress of air from the outside could be brought about. This can be done by the use of an air-shaft, made of wood or iron, and placed on a level with the floor, coming from the outside of the building. The end that enters the stable should be turned up a few inches, thereby avoiding all danger of draught and giving perfect ventilation. The entrance of air through this shaft should be controlled by a slide suitable to the conditions required.

In the November number of the JOURNAL the theory advanced by Dr. Claussen in regard to carbonic-acid gas is correct; but he does not go far enough in his argument, as in this matter we have the carbonic poison produced by and from the animal; being exhaled in a warm state or condition, it ascends to the top of the room or stable, and if allowed to remain there a sufficient time to become cold, it then descends to the floor, through the law of the diffusion of gases. So with top ventilation and an air-shaft at the floor we must certainly get rid of these gases as fast as they are produced. Firedamp in mines, chokedamp in wells, cellars, and over lakes, etc., do not apply to this subject, being produced under different conditions. To prove this theory of ventilation I quote from Youman's *Chemistry*. The author says that by raising and depressing an open umbrella in a well you can do away with the stifling effects of the carbonic-acid gas by mingling it with the air. So by this method of ventilation we keep the air in motion and so do away with the effete matter that is liable to be carried back into the system and also furnish an abundance of fresh air to the inmates of the stable.

I give you these facts, as I know them to exist from practical experience, not theory alone. An exchange of ideas on these matters I deem of great importance to all interested.

Fraternally yours,

J. WALLACE MARSH, V.S.

SCHOHARIE, Nov. 17, 1895.

---

THE following letter was received by Dr. W. Horace Hoskins, President of the United States Veterinary Medical Association, at Des Moines on the afternoon of the second day, and with many others was only noted as received by the Secretary, owing to the lack of time for consideration of the programme as presented. In justice to the writer Dr. Hoskins takes this manner of presenting it, so that it may be read by all at a time when when it may receive more considerate thought:

DR. W. HORACE HOSKINS,
President U. S. V. M. A.

DEAR DOCTOR: I assure you that it is a great disappointment to me to have to write this letter on this Sunday evening just as

I should be leaving here on the latest possible train which would land me in Des Moines in time for the meeting. I fully appreciate the loss which must result to me from my inforced absence. Those who are not in the habit of attending our meetings cannot know the benefit derived from them, both intellectually and socially, but I, as one who has never missed a meeting during ten years' membership, know full well that such a loss can hardly be estimated. I was particularly anxious to be present this year in order to converse informally with the members present as to the feasibility of dividing the meeting geographically into two or possibly three sections—the East, the Central, and the Western, each to be presided over by its vice-president in the absence of the president. The expenses of the president, secretary, and members of the comitia minora to be paid when outside of this section. That this meeting of the comitia minora should be held just before the meeting of the Central section, which meeting in its turn should be held before the meetings of the Eastern and Western sections. Matter, affecting the well-being of the Association as a whole would have to be submitted to them before being acted upon by the different sections. This idea may seem visionary and impracticable, but to me it is a plan which would create better fellowship and increase more the usefulness of our society than the present system of expecting a fair attendance of members from the far West and South, when most of the meetings have been and for a long time probably will be held in the East.

Certainly, we all expect to go to California as a body before any geographical sections are thought of. We want to get acquainted with our colleagues in the West and far West and the South and Southwest first, but it is just as well to talk matters over freely now. Nine days altogether of our annual meeting—three days for each section, with an intersectional congress, so to speak, once in three years—would, I believe, enthuse membership; so increase the amount of money in our treasury as to enable us to allow mileage to the officers of our Association and members of comitia minora when outside their section; and enable us to print proceedings, which would be of inestimable value to the veterinary world. The first secretary should be elected for a term of three years, with as much salary as we could afford to pay him. The length of term should apply to the president, that is, from one intersection congress to the next. Then let each section elect or name their vice-president.

I know that any such suggestion would come with better grace from me were I present, but my heart is with you, and it is only on account of the most unavoidable circumstances that I am absent in body.

Yours fraternally,

A. W. Clement.

Baltimore, September 8, 1895.

